# Chronic occupational exposure to hexavalent chromium causes DNA damage in electroplating workers

**DOI:** 10.1186/1471-2458-11-224

**Published:** 2011-04-12

**Authors:** Xu-Hui Zhang, Xuan Zhang, Xu-Chu Wang, Li-Fen Jin, Zhang-Ping Yang, Cai-Xia Jiang, Qing Chen, Xiao-Bin Ren, Jian-Zhong Cao, Qiang Wang, Yi-Min Zhu

**Affiliations:** 1 Hangzhou Center for Disease Control and Prevention, Hangzhou 310021, PR China; 2 Department of Epidemiology and Biostatistics, Zhejiang University School of Medicine, 388 Yu-Hang-Tang Road, Hangzhou 310058, Zhejiang, PR China; 3 Zhejiang Center for Disease Control and Prevention, Hangzhou 310051, PR China

## Abstract

**Background:**

Occupational exposure to chromium compounds may result in adverse health effects. This study aims to investigate whether low-level hexavalent chromium (Cr(VI)) exposure can cause DNA damage in electroplating workers.

**Methods:**

157 electroplating workers and 93 control subjects with no history of occupational exposure to chromium were recruited in Hangzhou, China. Chromium levels in erythrocytes were determined by graphite furnace atomic absorption spectrophotometer. DNA damage in peripheral lymphocytes was evaluated with the alkaline comet assay by three parameters: Olive tail moment, tail length and percent of DNA in the comet tail (tail DNA%). Urinary 8-OHdG levels were measured by ELISA.

**Results:**

Chromium concentration in erythrocytes was about two times higher in electroplating workers (median: 4.41 μg/L) than that in control subjects (1.54 μg/L, *P *< 0.001). The medians (range) of Olive tail moment, tail length and tail DNA% in exposed workers were 1.13 (0.14-6.77), 11.17 (3.46-52.19) and 3.69 (0.65-16.20), and were significantly higher than those in control subjects (0.14 (0.01-0.39), 3.26 (3.00-4.00) and 0.69 (0.04-2.74), *P *< 0.001). Urinary 8-OHdG concentration was 13.65 (3.08-66.30) μg/g creatinine in exposed workers and 8.31 (2.94-30.83) μg/g creatinine in control subjects (*P *< 0.001). The differences of urinary 8-OHdG levels, Olive tail moment, tail length and tail DNA% between these two groups remained significant (*P *< 0.001) even after stratification by potential confounding factors such as age, gender, and smoking status. Chromium exposure was found to be positively associated with chromium levels in erythrocytes, urinary 8-OHdG levels, Olive tail moment, tail length and tail DNA%. Positive dose-response associations were also found between chromium levels in erythrocytes and Olive tail moment, tail length and tail DNA%.

**Conclusion:**

The findings in this study indicated that there was detectable chromium exposure in electroplating workers. Low-level occupational chromium exposure induced DNA damage.

## Background

Chromium (Cr) is one of the eight metals in the top 50 priority list for toxic substances by the Agency for Toxic Substances and Disease Registry (ATSDR 2003). The majority of chromium in the environment exists in two valence states: trivalent chromium Cr(III) and hexavalent chromium Cr(VI) [1]. Cr(III) is generally benign due to poor membrane permeability [2]. Cr(III) compounds are even recognized as essential micronutrients that are involved in important physiological functions, such as the biological activity of insulin [3].

In contrast, Cr(VI) compounds can actively penetrate cell membrane through channels for isoelectric and isostructural anions, such as SO_4_^2- ^and HPO_4_^2- ^channels [4,5]. Insoluble chromates are uptaken via phagocytosis [6]. Cr(VI) is a strong oxidizing agent, and can be reduced through short-lived Cr intermediates (Cr(V) and Cr(IV)) to the stable trivalent state. The reactions of Cr(VI) with biological reductants, such as ascorbate and thiols, often generate free radicals, which in turn activate O_2 _and produce reactive oxygen species (ROS), including hydroxyl radicals, singlet oxygen, superoxide and hydrogen peroxide [7-10]. The resulting excessive production of ROS may lead to oxidative stress, damaging DNA and proteins [11]. Common forms of DNA damage include DNA strand breaks, chromium-DNA adducts, DNA-DNA and DNA-protein cross-links as well as oxidative DNA damage [12-16]. Due to these mutagenic properties, Cr(VI) is considered as a group 1 human carcinogen by the International Agency for the Research on Cancer [17,18]. Chronic exposure to Cr(VI) significantly increases the risk of respiratory tract cancer [18].

Occupational exposure to chromium occurs mainly through inhalation and dermal absorption in the working environment, including chromium compound manufacturing, electroplating, leather tanning, and welding. Previous studies on Cr(VI) toxicity mainly focused on relatively high-level chromium exposure, such as chrome-plating, chromium compound manufacturing and polishing [19-21]. Recent studies on exposure to relatively low levels of chromium such as at electroplating and tannery workplace have yielded inconsistent results [22,23]. The discrepancy might stem from small sample size and unadjusted confounding effects. In this study, in order to evaluate the potential health concerns related to chronic low-level chromium exposure, 157 electroplating workers and 93 control subjects were recruited and their chromium levels in erythrocytes were measured. DNA damage in peripheral lymphocytes was evaluated by alkaline single cell gel electrophoresis (the comet assay). The level of 8-hydroxydeoxyguanosine (8-OHdG) in urine, an indicator of oxidative stress, was measured with an enzyme-linked immunosorbent assay (ELISA) kit.

## Methods

### Study subjects

A total of 157 electroplating workers were recruited from 20 electroplating factories in Hangzhou, China from 2009 to 2010 and 93 control subjects were recruited from workers who were not occupationally exposed to chromium compounds or any other known physical or chemical genotoxic agents. All subjects were investigated for information of age, smoking habits, alcohol consumption, medical history and years of chromium exposure. Subjects with abnormal liver or kidney function and/or suffering from other chronic diseases such as cancer, diabetes and heart disease were excluded from the study. The study protocol was approved by the Institutional Review Board of Hangzhou Center for Disease Control and Prevention. Written informed consent was obtained from all subjects.

### Air sampling

Short-term sampling was conducted according to Specifications of Air Sampling for Hazardous Substances Monitoring in the Workplace in China (GBZ159-2004). Air samples were collected at the breathing zone and at the electroplating workplace. Nucleopore filter (diameter of 0.8 μm) was loaded in the filter cassette (diameter of 40 mm). The flow rate of air was set at 5 L/min and the sampling period was 15 min. Airborne chromium concentration was measured by graphite furnace atomic absorption spectrophotometer (AAS).

### Blood and urine collection

All sample containers were washed and rinsed overnight with 10% nitric acid to prevent contamination of chromium or other heavy metals. The peripheral vein blood sample (4 ml) was collected from each subject in the morning, and drawn into two vacuum tubes containing 3.6 mg of EDTA. One tube of blood (2 ml) was stored at 4°C for determining chromium levels in erythrocytes and the comet assay. The other tube of blood was stored at -80°C for DNA isolation and genotyping. The urine sample (2 ml) was obtained after a continuous working day. After measuring urinary creatinine concentration, the urine sample was stored in a nitric acid-treated polypropylene container at -20°C until analyzed for 8-OhdG concentration. All the samples once collected were kept on ice and delivered within the same day to the laboratory with minimal vibration.

### Measurement of chromium concentration in erythrocytes with graphite furnace AAS

The blood sample was centrifuged for 10 min at 2000 rpm to isolate erythrocytes. The erythrocytes were washed with phosphor-buffered saline (PBS) for three times. Chromium concentrations in erythrocytes were measured by graphite furnace AAS (Thermo M6) with Zeeman background correction. The standard curve was fitted with linear least-squares method. The detection limit of chromium was 0.2 μg/L. The recovery of standard addition was 95-98.8%. The concentration of chromium in erythrocytes was corrected for hematocrit for each subject.

### Determination of urinary 8-OHdG concentration

The competitive inhibition enzyme immunoassay technique was employed. Each urine sample was centrifuged at 1500 rpm for 10 min, and the supernatant was used for measuring 8-OHdG concentration. The concentration of 8-OHdG was determined by an ELISA kit following manufacturer's instructions (Cusabio, China). The concentration was adjusted by urinary creatinine levels and expressed as μg 8-OHdG/g creatinine.

### Comet assay

The alkaline comet assay was performed as previously described [24] with some modifications. Peripheral blood (10 μl) was mixed with 75 ul of 0.75% low-melting- point agarose and transferred to a microscope slide precoated with a layer of 0.75% normal-melting-point agarose. The slides were immersed in the lysis buffer (2.5 mol/L NaCl, 100 mmol/L EDTA, 10 mmol/L Tris, freshly added 1% Triton X-100 and 10% DMSO, pH 10) for 1 h at 4°C to remove proteins. The slides were then placed in a horizontal electrophoresis tank containing electrophoresis buffer (300 mmol/L NaOH, 1 mmol/L EDTA, pH 13) for 20 min to allow DNA unwinding. The electrophoresis was carried out in the same buffer for 20 min. After electrophoresis, the slides were neutralized in the neutralization buffer (0.4 mol/L Tris, pH 7.5), and then stained with 50 μL ethidium bromide solution (20 μg/mL). All the steps were conducted under yellow light to prevent additional DNA damage. One hundred nuclei were selected randomly from each sample. The observation was made at 400 × magnification using a fluorescence microscope (DMI 4000) equipped with a 530-nm excitation filter and a computer-based image analysis program CASP. The medians of Olive tail moment, tail length and tail DNA% were used for assessing DNA damage. Olive tail moment is defined as the product of the tail length and the fraction of total DNA in the tail and calculated as [(tail mean - head mean) × (tail DNA%/100)] [24].

### Statistical analysis

The median (range) was used to describe the average and variation for quantitative data. Analysis of variance (ANOVA) was employed to compare the differences between groups for normally distributed and homogeneous data. Nonparametric test (Kruskal-Wallis test) was used for non-normally distributed or heterogeneous data. Chi square test was used to compare count data. Multivariate linear model was used to modify the potential confounding effects. A *P *value of less than 0.05 was considered statistically significant. All statistical calculation was performed by SPSS 13.0.

## Results

### Demographic and occupational surveillance data

The demographic information of electroplating workers and control subjects is presented in Table 1. There were no significant differences in age, gender, smoking status, alcohol consumption between the two groups (*P *values > 0.05). Of all the exposed workers in this study, 95% wore gloves, 63% usually wore protective clothes, and 52% wore masks during their working shifts. Six percent of the exposed workers had nasal septum ulcer or a skin rash.

**Table 1:** Demographic information of the electroplating workers and control subjects.

variables	exposed workers	control subjects	***P *value**^**a**^
		
	n = 157	n = 93	
gender			0.47
male	101	64	
female	56	29	
age (years, mean ± sd)	39.7 ± 8.3	38.8 ± 9.6	0.47
smoking status^b^			0.18
yes	64	46	
no	93	47	
alcohol consumption^c^			0.18
yes	78	38	
no	79	55	
years of chromium exposure (median (min-max))	5.3(0.5-23)	--	

^a ^*P *values were calculated from Pearson's χ^2 ^test for categorical variables (gender, smoking status, alcohol consumption) and Student's t-test for age.

^b ^Smoking status was defined as: ≧one cigarette per day for more than one year or quit smoking for less than one year.

^c ^Alcohol consumption was defined as: at least once a week for more than six months.

The median of short-term exposure concentration of chromium in the air at electroplating factories was 0.060 mg/m^3 ^(ranging from 0.016 to 0.531 mg/m^3^), which was higher than the permissible concentration of short term exposure limit (PC-STEL) of chromium in China (0.05 mg/m^3^). Fifty-two percent of the factories investigated had chromium concentration above PC-STEL.

### The chromium concentration in erythrocytes (μg/L) in electroplating workers and control subjects

Chromium concentrations in erythrocytes (μg/L) in electroplating workers and control subjects are shown in Table 2. In electroplating workers, the median of chromium concentration in erythrocytes was about two times higher than that in control subjects (*P *< 0.001). After stratification by potential confounding factors such as gender, age, smoking status and alcohol consumption, significant differences remained between exposed workers and control subjects except for the subjects less than 30 years old (*P *= 0.11). In addition, among electroplating workers, the chromium concentration of erythrocytes in smokers was significantly higher than that in non-smokers (*P *< 0.05).

**Table 2:** Chromium concentration in erythrocytes in the electroplating workers and control subjects.

variables	chromium in erythrocytes (μg/l)		***P *value**^**a**^
		
	exposed workers	control subjects	
overall	4.41(0.93-14.98)	1.54(0.14-4.58)	<0.001
gender			
male	4.30(0.93-14.98)	1.29(0.14-4.58)	<0.001
female	4.63(1.18-13.70)	1.88(0.84-4.21)	0.002
*P *value^b^	0.72	0.26	
age			
<30	3.93(0.93-12.86)	1.44(0.14-3.58)	0.11
30-40	4.77(1.10-14.98)	1.65(0.17-3.30)	0.002
>40	4.26 (1.07-13.00)	1.68(0.29-4.58)	<0.001
*P *value^b^	0.41	0.64	
smoking status			
no	3.58(0.93-12.64)	1.29(0.17-4.58)	0.001
yes	4.98(1.18-14.98)	1.85(0.14-4.21)	<0.001
*P *value^b^	0.008	0.57	
alcohol consumption			
no	4.14(1.07-12.78)	1.44(0.14-4.21)	0.006
yes	4.69(0.93-14.98)	1.79(0.17-4.58)	<0.001
*P *value^b^	0.75	0.72	

Data were presented as median (range);

*P *values were calculated with rank sum test of chromium concentration in erythrocytes. ^a ^between the electroplating workers and control subjects; ^b ^between potential confounding factors in the electroplating workers, control subjects, respectively.

### The Olive tail moment, tail length, tail DNA% and urinary 8-OHdG concentration (μg/g creatinine) in electroplating workers and control subjects

Summarized in Table 3, the medians of Olive tail moment, tail length and tail DNA% in electroplating workers were significantly higher than those in control subjects (*P *< 0.05). In addition, urinary 8-OHdG concentration in electroplating workers was also significantly higher than that in control subjects (*P *< 0.05).

**Table 3:** Urinary 8-OHdG (μg/g creatinine), Olive tail moment (OTL), Tail Length and Tail DNA% in the electroplating workers and control subjects.

variables	8-OHdG			**OTL**^**#**^			Tail Length			Tail DNA%		
	
	exposed workers	control subjects	***P *value**^**a**^	exposed workers	control subjects	***P *value**^**a**^	exposed workers	control subjects	***P *value**^**a**^	exposed workers	control subjects	***P *value**^**a**^
overall	13.65 (3.08-66.30)	8.31 (2.94-30.83)	<0.001	1.13 (0.14-6.77)	0.14 (0.01-0.39)	<0.001	11.77 (3.46-52.19)	3.26 (3.00-4.00)	<0.001	3.69 (0.65-16.20)	0.69 (0.04-2.74)	<0.001
gender												
male	13.10 (3.08-66.30)	7.51 (2.94-22.34)	<0.001	1.25 (0.14-6.77)	0.14 (0.03-0.39)	<0.001	13.09 (3.46-52.19)	3.26 (3.00-4.00)	<0.001	4.05 (0.74-16.20)	0.69 (0.10-2.74)	<0.001
female	14.71 (3.49-33.90)	9.95 (3.55-30.83)	0.006	0.90 (0.16-3.52)	0.11 (0.01-0.23)	0.002	9.43 (3.72-26.97)	3.25 (3.00-4.00)	<0.001	3.05 (0.65-11.17)	0.67 (0.04-1.20)	<0.001
*P *value^b^	0.41	0.06		0.07	0.68		0.011	0.964		0.065	0.974	
age												
<30	10.91 (4.51-27.28)	5.07 (3.55-7.11)	<0.001	0.79 (0.16-2.78)	0.07 (0.01-0.12)	0.003	8.80 (3.69-27.97)	3.20 (3.00-4.00)	<0.001	2.68 (0.65-8.02)	0.44 (0.09-1.11)	0.001
30-40	13.38 (3.49-59.15)	8.66 (2.94-22.34)	0.03	1.34 (0.14-6.77)	0.09 (0.05-0.27)	<0.001	13.09 (3.46-52.19)	3.26 (3.00-4.00)	<0.001	4.30 (0.74-16.20)	0.72 (0.13-1.74)	<0.001
>40	14.59 (3.08-66.30)	10.02 (3.30-30.83)	0.05	1.06 (0.24-4.26)	0.16 (0.08-0.39)	<0.001	11.59 (3.87-35.97)	3.27 (3.00-4.00)	<0.001	3.51 (1.19-12.74)	0.77 (0.04-2.74)	<0.001
*P *value^b^	0.52	0.001		0.06	0.67		0.038	0.947		0.082	0.493	
smoking status												
No	9.65 (3.08-32.32)	8.20 (2.94-22.34)	0.04	1.07 (0.16-6.77)	0.12 (0.01-0.27)	<0.001	11.19 (3.72-52.19)	3.11 (3.00-4.00)	<0.001	3.46 (0.65-16.20)	0.57 (0.04-1.32)	<0.001
Yes	16.38 (3.49-66.30)	8.40 (3.30-30.83)	<0.001	1.21 (0.14-4.26)	0.18 (0.03-0.39)	<0.001	12.64 (3.46-35.97)	3.33 (3.00-4.00)	<0.001	4.02 (0.74-12.74)	0.75 (0.13-2.74)	<0.001
*P *value^b^	<0.001	0.50		0.32	0.57		0.249	0.223		0.200	0.471	
alcohol consumption												
no	12.28 (4.09-59.15)	7.74 (2.94-30.83)	0.03	1.06 (0.16-6.77)	0.11 (0.01-0.23)	<0.001	10.95 (3.72-52.19)	3.19 (3.00-4.00)	<0.001	3.44 (0.65-16.20)	0.68 (0.04-2.74)	<0.001
yes	15.12 (3.08-66.30)	9.22 (3.30-22.34)	<0.001	1.21 (0.14-4.26)	0.14 (0.05-0.39)	<0.001	12.73 (3.46-35.97)	3.36 (3.00-4.00)	<0.001	3.99 (0.74-12.74)	0.71 (0.22-1.74)	<0.001
*P *value^b^	0.05	0.57		0.16	0.46		0.064	0.314		0.159	0.430	

Data were presented as median (range);

# Olive tail moment.

*P *values were calculated with rank sum test. ^a ^between the electroplating workers and control subjects; ^b ^between potential confounding factors in the electroplating workers, control subjects, respectively.

After stratification by gender, age, smoking status and alcohol consumption, the differences in Olive tail moment, tail length, tail DNA% and urinary 8-OHdG concentration remained significant between the two groups (*P *< 0.05). Statistical significance was also found in the comparison of urinary 8-OHdG concentration between smoking and non-smoking electroplating workers and among different age groups in control subjects, as well as in the comparison of tail length between male and female electroplating workers and among different age groups of electroplating workers (*P *< 0.05). Borderline significance (0.05 <*P *< 0.1) was found in the comparison of urinary 8-OHdG levels between male and female control subjects, electroplating workers with different alcohol consumption status, as well as in the comparison of Olive tail moment and tail DNA% between male and female electroplating workers and among different age groups of electroplating workers.

### Multivariate analysis of the associations of occupational chromium exposure with chromium concentration in erythrocytes, Olive tail moment, tail length, tail DNA% and urinary 8-OHdG concentration

Multivariate linear models were used to study the associations of occupational chromium exposure with chromium concentration in erythrocytes, Olive tail moment, tail length, tail DNA% and urinary 8-OHdG levels after adjusting for potential confounding factors. After controlling for gender, age, smoking status and alcohol consumption, the occupational chromium exposure was positively associated with the chromium concentration in erythrocytes, Olive tail moment, tail length, tail DNA% and urinary 8-OHdG concentration (*P *< 0.001, summarized in Table 4). The chromium concentration in erythrocytes was positively associated with Olive tail moment, tail length and tail DNA% (*P *< 0.05). However, no significant association was found between chromium concentration in erythrocytes and urinary 8-OHdG levels (Table 5).

**Table 4:** Effect of occupational chromium exposure on chromium in erythrocytes, Urinary 8-OHdG, Olive tail moment, Tail Length and Tail DNA%.

dependent variables*	chromium exposure		**r**^**2§**^
		
	**95%CI**^**#**^	*P *value	
chromium in erythrocytes	0.399 (0.421,0.964)	<0.001	0.242
urinary 8-OHdG	0.263 (0.321,0.989)	<0.001	0.191
Olive tail moment	0.591 (0.511,0.826)	<0.001	0.347
Tail Length	0.550(1.142,1.942)	<0.001	0.315
Tail DNA%	0.600(0.850,1.358)	<0.001	0.356

* The raw data of dependent variables (Cr (VI) in erythrocytes, Tail Length, Tail DNA%, Olive tail moment and urinary 8-OHdG) were transformed with square-root.

** Chromium exposure was quantified as 1 = exposed, 0 = un-exposed.

^# ^the linear regression coefficient.

^§ ^r^2^, the decisive coefficient in the equation.

∫ Adjusted factors were defined as gender: 0 = male and 1 = female; age: 0 =< 30, 1 = 30-40, 2 => 40; smoking status: 0 = no, 1 = yes; alcohol consumption, 0 = no, 1 = yes

**Table 5:** Effect of chromium concentration in erythrocytes on, Urinary 8-OHdG, Olive tail moment, Tail Length and Tail DNA%.

dependent variables*	**95%CI**^**#**^	*P *value	**r**^**2 §**^
urinary 8-OHdG	0.146 (-0.171,1.163)	0.143	0.157
Olive tail moment	0.445 (0.357,0.847)	<0.001	0.336
Tail Length	0.387(0.356,1.031)	<0.001	0.206
Tail DNA%	0.352(0.178,0.618)	0.001	0.154

* In the multivariate linear model, chromium concentration in erythrocytes, gender, age, smoking status, alcohol consumption were independent variables and Olive tail moment, Tail Length, Tail DNA% and urinary 8-OHdG were dependent variables, respectively. The raw data of dependent variables (Olive tail moment, Tail Length, Tail DNA% and Urinary 8-OHdG) were transformed with square-root.

^# ^the coefficient of linear regression.

^§ ^r^2^, the decisive coefficient in the equation.

## Discussion

Cr(VI) compound is a strong oxidizing agent and can lead to oxidative stress and DNA damage. While high-level chromium exposure has generally been considered to have carcinogenic effects [19-21], the health concerns regarding low-level chromium exposure have failed to reach a consensus [22,23]. Compared to industries with high-level chromium exposure such as chrome plating, electroplating is generally considered to have much lower-level chromium exposure [25]. Thus in this study, we investigated the effects of chronic low-level chromium exposure on DNA damage in electroplating workers. Chromium concentration in erythrocytes was used to monitor hexavalent chromium exposure. Urinary 8-OHdG was used to assess the cellular oxidative stress. Alkaline comet assay was used to determine DNA strand breaks and cross-links in lymphocytes. We also adjusted for confounding factors such as age, sex, smoking status and alcohol consumption using stratification and multivariate linear model. The results showed that chromium concentration in erythrocytes in electroplating workers was significantly higher than that in control subjects. The exposed workers had higher levels of Olive tail moment, tail length, tail DNA% in lymphocytes and higher urinary 8-OHdG concentration. Chromium exposure was positively associated with urinary 8-OHdG, Olive tail moment, tail length and tail DNA%. Together current results indicated that electroplating workers experienced chronic occupational Cr(VI) exposure, which induced oxidative stress and DNA damage.

Chromium exposure was traditionally evaluated by measuring chromium levels in blood and/or urine. However, urinary or blood chromium levels mainly reflect current or recent, rather than chronic cumulative, exposure to total chromium including Cr(III) and Cr(VI) [21]. Cr(III) is rapidly excreted into urine and is less toxic due to poor membrane permeability whereas membrane-penetrating Cr(VI) compounds induce DNA damage and carcinogenesis. Compared to the chromium levels in blood and urine, the level in erythrocytes which was used in the current study has better specificity for reflecting Cr(VI) exposure and the associated DNA damage, and thus serves as a better index [26,27]. Consistent with previous findings, chromium concentration in erythrocytes was significantly higher in electroplating workers than that in control subjects in this study. The value (4.4 μg/L) was also comparable to previously reported 4.3 μg/L in electroplating workers[25]. In comparison, higher chromium concentration (4.9 to 6.0 μg/L) has been reported in chrome-plating workers[19,21], probably due to higher-level chromium exposure. Further analysis showed that smoking had a significant effect on chromium concentration in erythrocytes in electroplating workers but not in control subjects (Table 2), indicating that smoking increased chromium burden in occupationally exposed individuals.

Recent studies reveal that chromium exposure can be genotoxic (ATSDR 2008). The present study showed in electroplating workers, Olive tail moment, tail length and tail DNA% were significantly higher than those in the control subjects even after controlling for gender, age, smoking status and alcohol consumption, indicating marked DNA damage in electroplating workers. These results were consistent with previous studies. Gambelunghe et al. [19] discovered that comet tail length, tail intensity and tail moment were all significantly increased in lymphocytes of chromium exposed workers. Lee et al. [28] found a 1.6-fold increase in tail DNA% in white blood cells of subjects after 1 h treatment of 100 μmol/L sodium dichromate. Danadevi et al. [29] revealed that for welders, the levels of Cr(VI) in the workplace correlated positively with the DNA damage in peripheral blood leukocytes.

Cr(VI) also induces the formation of 8-OHdG, one major oxidative adduct induced by radical damage to DNA [30]. Measuring urinary 8-OHdG as an index of oxidative stress in cells has been commonly used because it is non-invasive and easy to perform. In this study, we found urinary 8-OHdG concentration of electroplating workers was significantly higher than that of control subjects even after stratification for confounding factors, which was consistent with a previous study [31].

The associations between non-occupational factors and urinary 8-OHdG levels have been inconclusive. Previous findings revealed significant correlations between smoking and urinary 8-OHdG [32,33], in which urinary 8-OHdG reflected oxidative damage in lung tissue due to smoking. But two recent reports failed to replicate the association [34,35]. In the current study, smoking was only significantly associated with urinary 8-OHdG levels in electroplating workers. In addition, there have been inconsistent reports on the association of urinary 8-OHdG levels with age [31,34,36]. In this study, urinary 8-OHdG levels were positively correlated with age only in control subjects but not electroplating workers. Furthermore, the estrogen level also plays an important role in gender differences of urinary 8-OHdG levels [34,35,37]. In this study, the difference between male and female only reached borderline significance for control subjects. The many controversies discussed here might stem from relatively small sample size, different subject background and experience, and lack of control for additional confounding factors, such as individual's ability to repair DNA [38]. Those issues might have distorted the statistical analysis and resulted in the discrepancies across different studies.

The electroplating workers might also be exposed to other metals, such as nickel, zinc and copper, besides chromium at the workplace. We also looked into this issue and measured the concentration of airborne soluble nickel. We found the level was generally below the detectable limit except for one sample [mean: 0.033 mg/m^3^, far lower than the PC-STEL of soluble nickel in China (0.5 mg/m^3^)]. Chromic anhydride can be evaporated into air and airborne chromium can be detected at the electroplating workplace. With the protection of gloves, apron and boots, respiratory system is the main way through which potential toxic metals are absorbed into the body. Therefore, chromium compounds may be the main source of metal exposure for electroplating workers and the major contributor to DNA damage and oxidative stress.

## Conclusion

The findings of this study indicated that there was higher level of occupational chromium exposure and detectable DNA damage in electroplating workers. DNA damage was associated with the chromium level in erythrocytes, which may serve as a useful biomarker for hexavalent chromium.

## List of abbreviations

8-OHdG: 8-hydroxydeoxyguanosine; Cr(III): trivalent chromium; Cr(VI): hexavalent chromium; PC-STEL: permissible concentration short term exposure limit; AAS: atomic absorption spectrophotometer.

## Competing interests

The authors declare that they have no competing interests.

## Authors' contributions

XZ participated in the epidemiological investigation, performed comet assay and ELISA, analyzed data and drafted the manuscript. XHZ was a chief investigator and was responsible for the epidemiological design, and sample collection and drafting the manuscript. XHZ and XZ contributed equally to this work. LFJ performed determination of chromium concentration in erythrocytes. ZPY, CXJ, XCW and JZC carried out the health surveillance in the workplace and sample collection. QC and XBR coordinated the study in field. YMZ participated in the overall design, study coordination, data analysis, and finalized the draft of the manuscript. All the authors read and approved the final manuscript.

## Pre-publication history

The pre-publication history for this paper can be accessed here:

http://www.biomedcentral.com/1471-2458/11/224/prepub
